# Heavy Metals in Iron Tailing Around River Sediments of Xiangshan: Status, Risks, and Human Health Threats

**DOI:** 10.3390/toxics14040284

**Published:** 2026-03-27

**Authors:** Jun Chen, Guangcheng Xiong, Shutong Zhang, Xianghui Lv, Qiang Tang, Qiuhong Zhou

**Affiliations:** 1 China National Chemical Construction Investment Group Co., Ltd., Beijing 102300, China; 2 Changjiang Survey, Planning, Design and Research Co., Ltd., Wuhan 430010, China; 3 Hubei Provincial Engineering Research Center for Comprehensive Water Environment Treatment in the Yangtze River Basin, Wuhan 430010, China; 4 Key Laboratory of Yangtze River Management and Protection of Ministry of Water Resources, Wuhan 430010, China; 5 School of Resources and Civil Engineering, Gannan University of Science and Technology, Ganzhou 341000, China

**Keywords:** iron tailings area, river sediments, heavy metal, source appointment, ecological risk

## Abstract

The heavy metal pollution linked to extractive activities has attracted broad public attention. To examine the current state of heavy metal pollution in river sediments around iron tailing zones, this study was carried out to evaluate the distribution features, potential sources, and environmental hazards of heavy metals (HMs, Cr, Cd, Ni, Cu, Zn, Pb, As, and Hg) in the surface sediments of rivers in the Xiangshan area of Ma’anshan City. Results indicated that, except for Cr, the mean heavy metal concentrations exceeded the soil background levels in Anhui’s Huaihe River Basin. Variability in metal concentrations among the sediments was moderate, exhibiting an uneven spatial distribution. Significant positive correlations were detected between various HMs in the sediments, suggesting a common pollution source. Source analysis findings revealed that the HMs primarily originate from agricultural fertilization, mining, and smelting activities. Evaluation results from both the single-factor pollution index and the Nemerow comprehensive index indicated that the upstream section of the Caishi River is severely polluted by HMs. The potential ecological risk index evaluation results demonstrated that 85% of sediment samples from sampling points achieved a high comprehensive potential ecological risk level for HMs, with Cd, Cu, and Hg identified as the key contributors. The human health risk assessment demonstrated that both adults and children are subjected to carcinogenic risks from heavy metal exposure, with children exhibiting a higher risk level. This study offers valuable insights into managing heavy metal contamination in river sediments adjacent to iron tailings regions.

## 1. Introduction

The development of mineral resources has significantly promoted social and economic progress. The inadequate management of mining waste poses a significant environmental threat. It can contaminate nearby farmland, surface water, and groundwater [1]. Sulfide minerals in the tailings area react with water under specific conditions to form sulfuric acid, creating acid mine drainage (AMD) that contains large amounts of heavy metal pollutants [2]. Driven by runoff, HMs from the tailings area migrate into the local soil and aquatic systems. These contaminations adversely affect the surrounding ecosystem and could enter the food chain, posing risks to public health [3]. Therefore, studying the current status of heavy metal pollution in the environment around the tailings area is of great significance.

Heavy metal pollution in the environmental media around mining areas has attracted widespread attention globally. Numerous studies have reported the characteristics, spatial distribution, ecological risks, and health risks associated with heavy metal contamination in soil [4], water bodies [5], and farmland crops [6] around mining areas worldwide. The 2014 National Soil Pollution Status Bulletin pointed out that in mining areas, about 33.4% of the soil exceeded the standard metal content, with the main inorganic pollutants being Cd, As, Zn, and Pb [7]. Chen et al. [3] performed an investigation of heavy metal contamination in the rainwater runoff of the stibium mining region in Lengshuijiang City, Hunan Province, China. Their findings revealed that toxic heavy metal runoff from these mining areas can severely degrade water quality and threaten the health of local communities. An assessment of heavy metal pollution and associated health threats in the farmlands of the world’s most extensive barium mining district was conducted by Liu et al. [8]. Their results indicated that agricultural soils were severely contaminated with cadmium (Cd) and arsenic (As), with barium mining and smelting activities identified as the primary sources of these HMs. These contaminants pose dual threats to local communities, with both carcinogenic and toxic effects identified as significant health concerns in the study area. Wang et al. [9] investigated heavy metal contamination of river sediments from a polymetallic mining region in Tonglushan, Daye City, Hubei Province, China. Their results demonstrated extremely high levels of heavy metal contamination in the sediment, along with a significant ecological risk, as 64% of the sampled sites exhibited severe hidden bio-toxic effects. Additionally, Chris et al. evaluated the potential ecological risks of heavy metals in water, sediments, and shellfish along the Opuroama Creek in the Niger Delta, Nigeria, and found that the heavy metals in sediments posed a significant ecological risk, particularly with Cd [10]. Dusengemungu et al. assessed the concentration of heavy metals in soil from mine waste dumpsites around Kitwe and Mufulira, Zambia [11]. The results showed that the soil surrounding the copper tailings dam is severely contaminated with heavy metals [11]. Although the issue of heavy metal contamination of farmland and water bodies surrounding tailings areas has attracted widespread attention [12,13,14], the contamination of adjacent river sediments remains relatively understudied. As an important component of river ecosystems, sediments accumulate large amounts of nutrients, organic matter, and heavy metal pollutants due to long-term adsorption and deposition, becoming the primary source of endogenous pollution in water bodies [15,16,17]. The primary sources of HMs in water sediments are mining, smelting, and other industrial and agricultural activities. Long-term mining activities have intensively contaminated surrounding rivers with HMs through wastewater discharge, causing significant accumulation in the sediments. These HMs pose a threat to the surrounding ecosystem and ultimately to human health via the food chain [18]. Therefore, sediment pollution in riverbeds is an issue that cannot be overlooked in the management and restoration of river ecosystems [19]. Clarifying the pollution characteristics of HMs and other contaminants in sediments and their ecological and environmental risks is a crucial prerequisite for effective sediment pollution management.

Ma’anshan City is situated in the lower Yangtze River region. The Xiangshan area in Ma’anshan City is abundant in iron, copper, and other mineral resources. It has a history of open-pit mining spanning nearly a century, which earned it the title “grain warehouse of Ma Steel.” However, this long-term mining, including large-scale open-pit ore extraction, ore processing, tailings stacking, and waste rock dumping, has caused a series of environmental issues. This research focuses on the rivers surrounding a typical metal tailing area in the Yangtze River Basin. The occurrence characteristics and ecological risks of HMs in river sediments were analyzed. The sources of HMs were further analyzed to pinpoint potential contamination inputs. The findings of this study serve as a key resource for evaluating pollution risks and guiding subsequent remediation of heavy metal-contaminated sediments in the region, as well as a reference for similar mining-affected regions worldwide.

## 2. Materials and Methods

### 2.1. Overview of the Study Area

The Caishi River, also known as the Waiqiao River, is a tributary of the Yangtze River. It is located in Ma’anshan City, Anhui Province, China. Situated in the north subtropical zone, it experiences a tropical monsoon humid climate with an average annual temperature of 15.6 °C and an average annual precipitation of 1070 mm. The river originates from the Xiangshan area and flows east to west into the Yangtze River. This river is significant for Ma’anshan City, contributing to urban stormwater management, ecological balance, and agricultural irrigation. The upper reaches of the Caishi River are situated in the Nanshan Mine area of the Maanshan Iron and Steel Company, serving both as an industrial water source for mining operations and as a confluence point for mining wastewater.

### 2.2. Chemicals and Regeants

Mixed standard solutions of soil heavy metals were purchased from the National Reference Material Resource Sharing Platform (Chaoyang District, Beijing, China). Standard solutions of Cd, Cu, Zn, Cr, Pb, Ni, As, and Hg were purchased from the National Analysis and Testing Center for Nonferrous Metals and Electronic Materials (Huairou District, Beijing, China). All these heavy metal standard solutions had a concentration of 1000 μg/L. During the test, they were diluted to different concentrations with 1% nitric acid solution to draw the standard curve. Other reagents used in this study, such as HCl, HNO_3_, and HF, were of superior grade purity and purchased from Wuhan Xinshen Chemical Technology Co., Ltd. (Wuchang District, Wuhan, China). The laboratory water was deionized water prepared by a water purifier purchased from Sichuan Ulupure Technology Co., Ltd. (UPC-III-10T, Pidu District, Chengdu, China).

### 2.3. Sample Collection and Analysis

**Figure 1:**
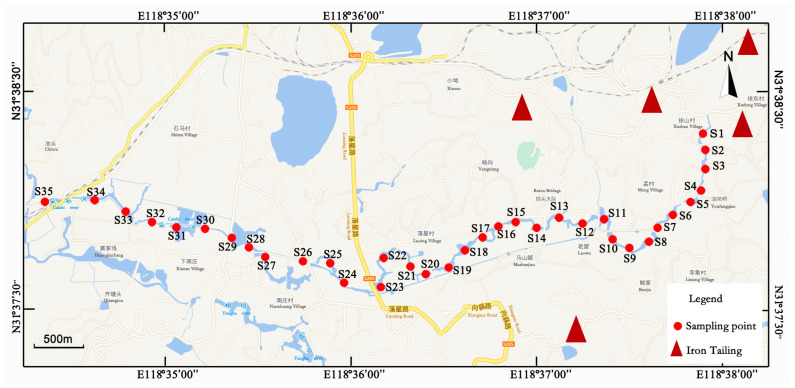
Distribution of the sediment sampling sites in the research area.

From June to July 2022, 35 sampling points were established along a 7.5 km section of the upper reaches of the Caishi River to collect surface sediments, as illustrated in Figure 1. Surface sediment samples from the 0–10 cm layer were collected. To ensure that the collected sediment samples can represent the actual pollution status of the sediment in the Caishi River, three samples were collected at each sampling site. After removing impurities such as animal and plant residues and gravel from the three sediment samples, they were mixed uniformly in a clean plastic box. About 2 kg of sample (for samples with high moisture content) was taken, placed in a self-sealing bag, and stored under refrigeration, then transported back to the laboratory within 24 h. The appearance and shape of the sediment were recorded simultaneously during sampling. In the laboratory, the samples were naturally air-dried at room temperature. And 50 g of each sample was weighed and ground with a mortar until all particles passed through a 200-mesh nylon sieve, then stored in a self-sealing bag.

Sediment samples were digested using a mixed acid solution (nitric acid–perchloric acid–hydrofluoric acid) [20]. The concentrations of heavy metals (Cd, Cu, Zn, Cr, Pb, and Ni) in the samples were determined using inductively coupled plasma mass spectrometry (ICP-5000, Focused photonics Inc, Binjiang District, Hangzhou, China), while arsenic (As) and mercury (Hg) were analyzed using atomic fluorescence spectrometer (AFS, F7100, HITACHI, Tokyo, Japan). To prevent sample contamination during analysis, one blank sample was analyzed for every ten samples. The results showed that the heavy metal contents in the blank samples were less than 0.001 of those in the tested samples. Additionally, two soil heavy metal mixed standard samples were included in each batch as quality control samples. The recovery rates of the quality control samples ranged from 86% to 117%, and the measurement errors were within ±8%, meeting the quality control requirements. To determine the organic matter (OM) content, sediment samples were subjected to loss on ignition in a muffle furnace (KSL-1200X, HF-Kejing, Hefei, China) at 550 °C for a duration of 4 h [21]. Sediment pH was determined by a glass electrode (PHSJ-5T pH meter, REX, Jiading District, Shanghai, China) after calibrating the instrument with standard buffer solutions (pH 4.6, 7.0, and 10.0). The measurement was conducted on a 1:2.5 (g/mL) sediment–water mixture.

### 2.4. Environmental Risk Assessment

The single-factor pollution index method (SFPI), the Nemerow comprehensive pollution index method (NPI), and the geo-accumulation index method (Igeo) were employed to analyze the pollution level of HMs in the sediment of the Caishi River [22,23]. Furthermore, the potential ecological risk index method (PERI) was used to evaluate the impact of heavy metals in the sediments of the Caishi River on the surrounding environment. The details are listed in the Supplementary Materials.

### 2.5. Human Health Risk Assessment

Human health risk assessment estimates the likelihood of harm to humans exposed to specific doses of chemical elements and proposes targeted protective measures to prevent chronic diseases. This study employs the human health risk assessment model recommended by the United States Environmental Protection Agency (EPA) to evaluate the health risks of residents near the Caishi River exposed to HMs in sediments. Specific calculation methods are listed in the Supplementary Materials [24], and relevant parameter selections are shown in Table 1 and Table 2.

### 2.6. Data Processing and Analysis

Statistical HMs concentration data was analyzed through Origin 2020 software. The SPSS 19.0 software was employed for the correlation analysis and principal component analysis in this study.

## 3. Results and Discussion

### 3.1. Analysis of Heavy Metal Concentration

Statistical analysis was performed on the concentrations of eight typical HMs (Cr, Cd, Ni, Cu, Zn, Pb, As, and Hg) in sediment samples from the Caishi River. The results are listed in Table 3. A significant variation in HM concentrations was observed in the river sediments, following the order: Cu (average: 384.28 mg/kg) > Zn (average: 180.67 mg/kg) > Cr (average: 47.19 mg/kg) > Ni (average: 42.03 mg/kg) > Pb (average: 28.85 mg/kg) > As (average: 11.80 mg/kg) > Cd (average: 1.07 mg/kg) > Hg (average: 0.10 mg/kg). Compared with the sediments of Chaohu Lake and the Anhui section of the Huai River [26,27], the study area exhibits higher concentrations of Cu and Zn. These findings were consistent with the pollution characteristics of HMs in soils adjacent to the tailing area [28]. It suggested that the high levels of Cu and Zn in the sediments were likely a result of prolonged and intensive mining operations.

Based on the soil background values in Anhui Province [22], the exceedance of Cd and Cu in the sediments is significant, with average exceedance multiples of 10 and 14 times, respectively. Similar to the present study, elevated concentrations of Cu, Zn, Cd, and As associated with historical mining activities have been widely reported in river basins influenced by metal tailings, including those in southern China, the Yangtze River Basin [29], and mining areas in North America and Europe [30]. The exceedance levels of Zn and Hg are lower, with average exceedance multiples of 3.4 and 2.4 times, respectively. The average exceedance multiples of Pb, Ni, and As are slightly above 1, while the average exceedance multiple of Cr is less than 1. These results indicated varying degrees of heavy metal enrichment in the river sediments, with notable enrichment of Cd and Cu, while Cr has did not show enrichment.

The coefficient of variation reflects the average degree of variation in heavy metal concentrations and can indicate the spatial variability of these heavy metal concentrations in the sampling area [31]. The coefficients of variation for HMs in the study area are moderate (0.1–0.9), suggesting some spatial heterogeneity in sedimentary metal concentrations. The coefficients of variation for Cu and Cd are relatively high (70% and 56%), indicating that the spatial discrepancies of Cu and Cd in the sediments are relatively sensitive and possibly impacted by local point sources.

The pH value and organic matter content of sediment are usually closely related to the content of heavy metals, so the pH and organic matter (OM) content of the 35 sediment samples were measured, and their statistical characteristics are summarized in Table S3. The pH values ranged from 6.6 to 7.7, with an average of 7.23 ± 0.35, indicating that the sediments in the study area were weakly alkaline overall. The OM content varied from 4.16% to 14.5%, with an average of 7.74 ± 2.58%, suggesting a moderate OM accumulation in the sediments, which is closely related to the input of organic matter from surrounding aquatic organisms, agricultural activities, and mining-related organic waste.

### 3.2. Source Analysis of HMs

#### 3.2.1. Correlation Analysis of HMs

**Figure 2:**
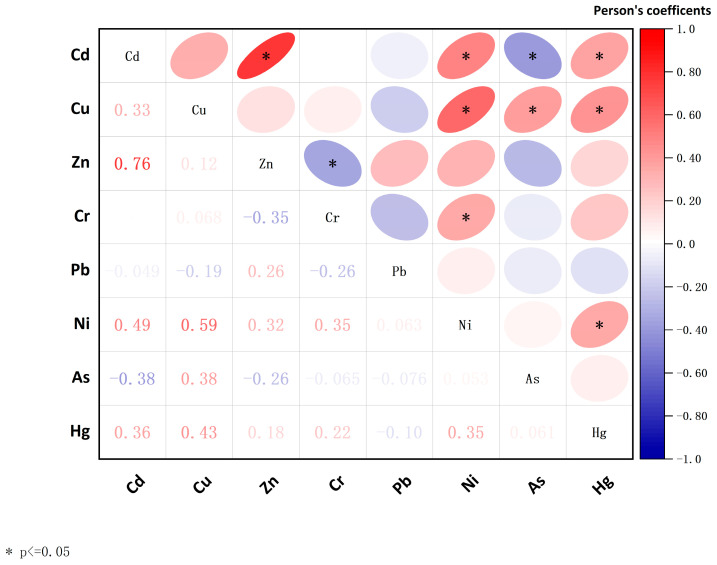
Correlation analysis of HMs in sediments.

Correlation analysis was conducted to investigate the relationships between different HMs. The results are illustrated in Figure 2. Cd and Zn exhibited a significant positive correlation, suggesting that they have similar origins, which might be determined by their geochemical origins [32,33]. Weaker positive correlations were also noted among other HMs, such as Cu and As, Cr and Ni, and Pb and As. Hg exhibited a significant weak correlation only with Cd and Cu, and no significant correlations with other HMs, likely due to its complex environmental behavior [34]. Although the correlations among different HMs suggest a certain degree of homogeneity in their sources, the sources of HMs warrant further analysis due to the complexity of their origins. To provide a more robust source identification, multivariate statistical analyses, such as principal component analysis (PCA) and Factor Analysis (FA), are widely employed as they can effectively reduce data dimensionality and reveal latent factors controlling pollutant variability [35].

Correlation analysis was further conducted to clarify the relationships between sediment pH, OM, and HM concentrations (Table S4). Correlation analysis revealed that pH exhibited moderate positive correlations with Cd (0.53) and Zn (0.57), indicating that elevated pH enhances the stability of these metals in sediments. This phenomenon is primarily attributed to the increase in hydroxide ions under slightly alkaline conditions, which promotes the adsorption and co-precipitation of Cd^2+^ and Zn^2+^ by sediment colloids, thereby reducing their release and facilitating enrichment in the sedimentary phase. Conversely, pH showed a moderate negative correlation with As (−0.41), suggesting that higher pH levels decrease As retention in sediments. Under alkaline conditions, arsenic predominantly exists as anionic species (e.g., AsO_4_^3−^ and AsO_3_^3−^), which are subject to electrostatic repulsion by negatively charged sediment colloids [36]. This results in reduced adsorption and enhanced leaching, consequently lowering As concentrations in sediments with increasing pH. Other metals, including Cu, Pb, Ni, Cr, and Hg, did not exhibit significant correlations with pH.

Organic matter (OM) demonstrated positive correlations with As, Ni, Cu, Hg, and Cr. This can be attributed to the abundant functional groups (e.g., -COOH and -OH) in sedimentary organic matter, which immobilize heavy metals through complexation, chelation, and adsorption [37]. Notably, OM exhibited a significant positive correlation with As (0.40), indicating that organic matter serves as a key factor controlling arsenic mobility in the study area. In contrast, OM showed weak correlations with Cd, Zn, and Pb, suggesting that the geochemical behavior of these metals is predominantly governed by other factors such as pH, mineral phases, and Fe-Mn oxides, rather than organic complexation.

#### 3.2.2. Principal Component Analysis of HMs

**Figure 3:**
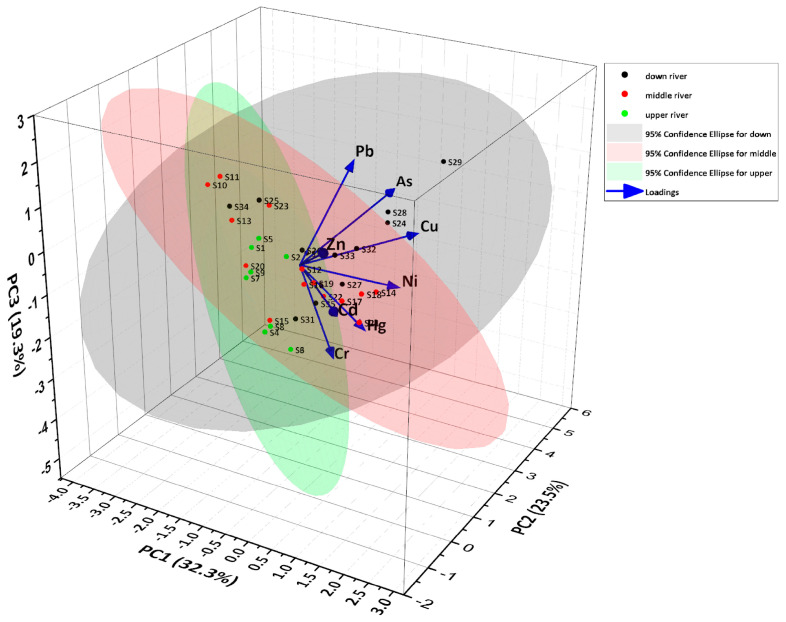
Principal component loading of HMs.

To identify the sources of HMs in the sediments, principal component analysis (PCA) was utilized (Figure 3). The PCA results indicate that three principal components with initial eigenvalues higher than 1 were extracted, accounting for a cumulative contribution rate of 85%, effectively explaining the original variables. In principal component 1, Cd and Zn exhibit higher loadings. Cd pollution is predominantly caused by agricultural activities, particularly through the application of phosphate fertilizers, which often contain Cd as an impurity due to the use of Cd-rich phosphate rock in their production [38]. Additionally, the use of certain pesticides and sewage sludge in agriculture can also contribute to Cd input into the soil environment. Given the presence of scattered farmlands near the sampling area, which are likely to receive regular fertilizer and pesticide applications, principal component 1 mainly represents agricultural pollution. Zn is an essential element for crop growth [39], and its elevated levels in this component may be attributed to agricultural practices such as the use of Zn-containing fertilizers and manure. In principal component 2, Cu, As, and Pb demonstrate higher loadings. Research indicates that mining and smelting operations are the primary sources of Cu, As, and Pb pollution [40]. The long-term pyrite mining activities upstream of the Caishi River contribute to the accumulation of Cu, As, and Pb in the sediments, making principal component 2 indicative of industrial pollution. In principal component 3, Cr and Hg show higher loadings. Cr is primarily derived from natural processes such as rock weathering, whereas Hg in areas without significant point source emissions is likely from atmospheric deposition [41,42]. Therefore, principal component 3 reflects atmospheric deposition sources. In summary, HM pollution of sediment in the study area was primarily linked to agricultural and industrial sources. They are the primary cause of contamination in river sediments around the tailing area. Natural sources, such as atmospheric deposition, have a limited effect.

These source apportionment results are consistent with recent findings from tailings-affected river systems worldwide, where mining activities are consistently identified as the dominant source of HMs. Studies using isotope tracing and multivariate statistics in multi-metal mining areas also confirm that tailing weathering and surface runoff are the primary pathways delivering HMs to river sediments [43]. Compared with watersheds dominated by a single pollution source, the Caishi River exhibits mixed source characteristics, which is typical of rivers surrounded by legacy tailings, farmland, and scattered villages. This pattern is representative of many small- to medium-sized rivers in mining regions of eastern China, where mixed land use and historical industrial legacies jointly shape sediment HM pollution profiles [43].

### 3.3. Ecological Risk Analysis of HMs

#### 3.3.1. Single-Factor Pollution Index Evaluation

**Figure 4:**
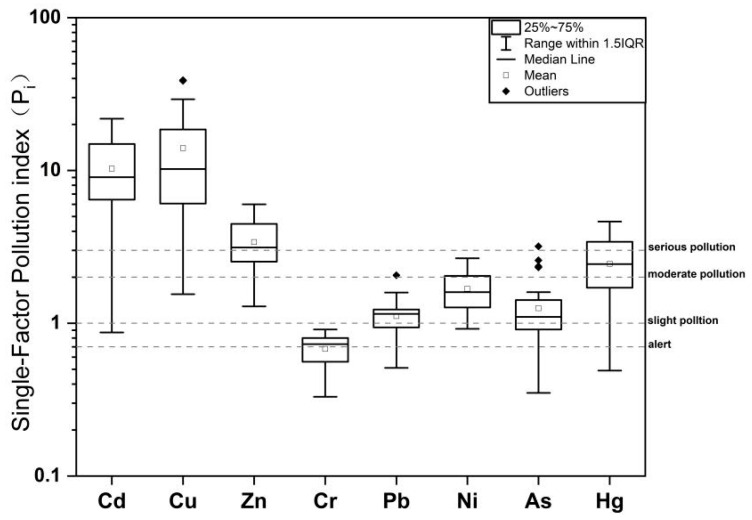
Single-factor index (*P_i_*) of HMs in sediments of the Caishi River.

The single-factor pollution index evaluation results of HMs in surface sediments of the Caishi River are listed in Figure 4. The results indicate that, except for Cr, there is a pollution risk for Cd, Cu, Zn, Pb, Ni, As, and Hg in the sediments (*P_i_* > 1). Among these metals, Cu is the most severely polluted, with 97% of sediment samples classified as severely polluted and an average *P_i_* value of 13.99. Cd is the second most severely polluted, with 94% of sediment samples classified as severely polluted and an average *P_i_* value of 10.27. In contrast, Zn and Hg exhibit relatively lower severe pollution proportions, at 63% and 37%, respectively. Except for one sediment sample where As reached severe pollution, the remaining sediments exhibit light-to-moderate pollution levels for As, Ni, and Pb. In abandoned mining areas, Cd commonly exhibits a high ecological risk due to its high toxicity coefficient and strong mobility, which aligns with our finding that Cd posed an extremely high ecological risk despite its relatively low total concentration.

#### 3.3.2. Nemerow Pollution Index Evaluation

**Figure 5:**
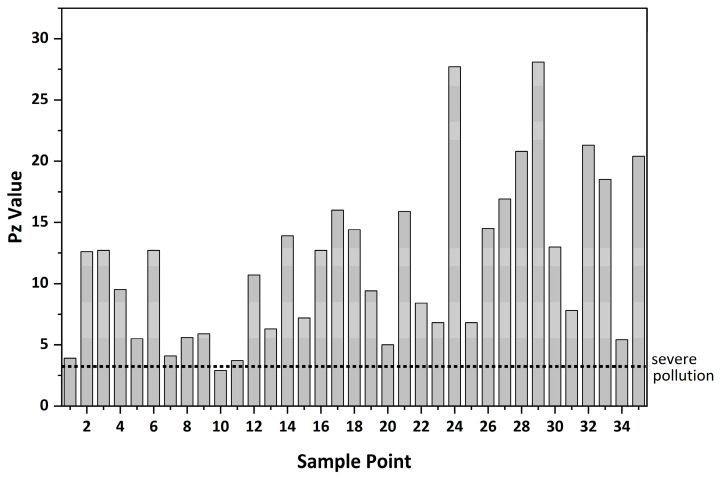
Comprehensive pollution index (*P_Z_*) of HMs in sediments of the Caishi River.

Figure 5 displays the assessment outcomes derived from the comprehensive pollution index regarding heavy metal contamination in the Caishi River’s surface sediments. Among the 35 sediment sampling points, 34 show severe pollution, accounting for 97.14%, while only sampling point No. 10 exhibits moderate pollution. Sampling point No. 29 is the most severely polluted with a *P_Z_* value of 28.1, followed by sampling point No. 24 with a *P_Z_* value of 27.7. Pollution levels are higher in the western part of the Caishi River than in the eastern part. Both the single-factor pollution index and the Nemerow composite pollution index evaluations indicate significant heavy metal pollution in the upstream sediments of the Caishi River. Consequently, attention should be paid to the potential pollution risks, and the dredged sediments should undergo harmless treatment. While the Nemerow composite pollution index is a widely used tool for integrating the effects of multiple pollutants, it is important to acknowledge its limitations. As noted in studies such as that by Li et al. (2023) [24], the results of such index methods can be sensitive to the choice of background values and the handling of extreme values. Therefore, to obtain a more comprehensive and robust assessment, it is beneficial to combine the Nemerow index with other evaluation methods, such as the geo-accumulation index and potential ecological risk index, as demonstrated in the literature [44].

#### 3.3.3. Geo-Accumulation Index Evaluation

**Figure 6:**
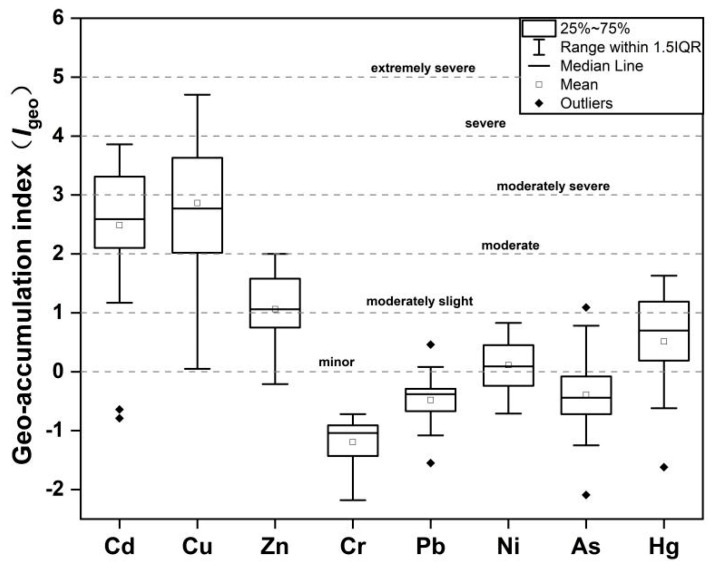
Geo-accumulation index (*I*_geo_) of HMs in Caishi River sediments.

Figure 6 shows the *I*_geo_ of HMs in the sediments of the study area. Significant differences in *I*_geo_ values are observed among different HMs, with pollution levels decreasing in the order of Cu > Cd > Zn > Hg > Ni > Pb > As > Cr. The *I*_geo_ ranges for Cu and Cd are 0.05–4.70 and −0.79–3.86, respectively, with maximum values indicating heavily and moderately polluted grades, suggesting higher pollution risks for these two metals. High pollution risks for Cu and Cd have also been reported in soils near pyrite mining areas in Anhui Province and iron mining areas in Gansu Province [45,46], indicating Cu and Cd as primary pollutants around the iron mining area. The remaining HMs in the study area range from clean to moderately polluted degrees, with Cr being clean but still having a higher *I*_geo_ than water environments without significant point source influences [47]. Acidic wastewater infiltration from mining areas and surface runoff may contribute to the heavy metal pollution in the surrounding river sediments [2].

#### 3.3.4. Potential Ecological Risk Index Evaluation

**Figure 7:**
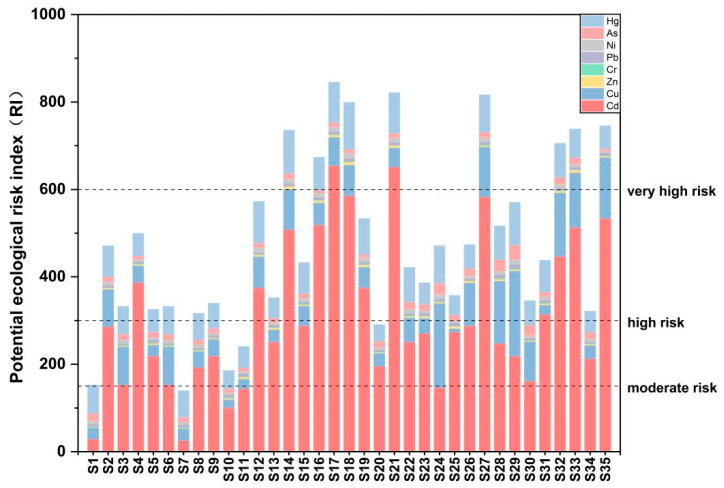
Risk index of HMs in Caishi River sediments.

The potential ecological risk index method, which integrates the toxicity coefficients of heavy metals, was applied to evaluate both the individual (E_r_^i^) and comprehensive (RI) ecological risk in the sediments of the study area. The calculation process of E_r_^i^ and RI of heavy metals in sediments in this study can be found in the Supplementary Materials. This method was originally proposed by Hakanson (1980) [48] and has been widely used in sediment ecological risk assessment. The toxicity coefficients (Tr) for each heavy metal (Cd = 30, Cu = 5, Zn = 1, Cr = 2, Pb = 5, Ni = 5, As = 10, Hg = 40) were adopted from the classic study by Hakanson (1980) [48], and the background values of heavy metals in sediments were derived from the soil background values of Anhui Province [7]. The analysis results indicate that the E_r_^i^ values of Cd, Cu, Zn, Cr, Pb, Ni, As, and Hg in the sediments are 28.85–654.81 (average: 307.99), 7.75–194.77 (average: 69.93), 1.29–6.00 (average: 3.34), 2.56–10.29 (average: 1.36), 7.75–194.77 (average: 5.57), 5.18–13.32 (average: 8.41), 3.52–31.86 (average: 12.56), and 47.55–106.58 (average: 67.24), respectively. Cd in the sediments posed a high-to-extremely high ecological risk. The average concentration of Cd in sediment is 1.07 mg/kg, which is 10 times higher than the soil background value. Additionally, its high toxicity contributes to the severe ecological risk posed by Cd in sediment. Cu and Hg in the sediments posed a moderate-to-very high ecological risk, while Zn, Cr, Pb, Ni, and As posed low risks. The results of the comprehensive potential ecological risk in the sediments indicate that the RI of HMs in the sediment of the Caishi River ranges from 139 to 822, with an average value of 476. This suggested that the ecological risk of HMs in river sediments was relatively serious, necessitating measures to prevent further environmental harm. The RI in the sediments samples shows a fluctuating trend from upstream to downstream of the river (Figure 7). Cd, Cu, and Hg are the HMs contributing the most to the potential ecological risk, with the contribution ratio of Cd in the sediment RI ranging from 19% to 79%. The surrounding area of the study is a tailing area, where long-term mining activities have led to the deposition of HMs in the river sediments via surface runoff. Additionally, scattered factories and villages around the study area may contribute to the high degree and spatial variation in heavy metal pollution in the river sediments.

### 3.4. Health Risk Assessment

Table 4 demonstrates the non-carcinogenic and carcinogenic risks of HMs in the sediment of the Caishi River through various exposure pathways. For adults, the non-carcinogenic risk indices for Cd, Cr, and Hg via oral intake and dermal contact are of the same order of magnitude, indicating that these are the primary exposure pathways for these metals. For Cu, Zn, Pb, and Ni, the non-carcinogenic risk values follow the order of oral intake > dermal contact > inhalation, suggesting that oral intake is the main exposure pathway for these metals. For As, the order is dermal contact > oral intake > inhalation. The HI values for adults exposed to the eight HMs follow the order: As > Cr > Pb > Cu > Cd > Ni > Zn > Hg. No significant non-carcinogenic risk to adults from individual heavy metals was found, as all HI values were below the threshold of 1. The THI value for adults is 0.3, suggesting relatively low non-carcinogenic health risks.

For children, the non-carcinogenic risk indices for oral intake of Cu, Zn, Cr, Pb, Ni, As, and Hg are 1–2 orders of magnitude higher than dermal contact and 3–5 orders of magnitude higher than inhalation, indicating that oral intake is the primary exposure pathway for these seven HMs. For Cd, the risk via oral intake and dermal contact is of the same order of magnitude. According to Table 4, children exhibit higher non-carcinogenic risk values than adults for all exposure routes. This pattern indicates their greater vulnerability to HM pollution. The HI values for children follow the order: As > Cr > Cu > Pb > Ni > Cd > Zn > Hg. All individual HI values are less than 1, indicating no significant non-carcinogenic risk to children from single HMs. The THI value for children is 1.01, indicating potential non-carcinogenic risks from multiple HMs in the sediment of the Caishi River, which relates to children’s behavior and physiological characteristics. Prior studies have demonstrated that children are more sensitive to environmental pollutants.

The carcinogenic risk assessment in this study was based on the slope factors (SFs) and reference doses (RfDs) recommended by the U.S. Environmental Protection Agency (USEPA, 2011, 2014) [49,50]. Specifically, the carcinogenic slope factors for Cd, Cr (VI), Pb, Ni, and As were derived from the USEPA Integrated Risk Information System (IRIS) database. Since no carcinogenic risk slopes were available for Cu, Zn, and Hg in the authoritative databases, this study only evaluated the carcinogenic risks of Cd, Cr, Pb, Ni, and As. The results are shown in Table 4. Both adults and children in the study area face carcinogenic risks, with total risk values exceeding the 1 × 10^−4^ threshold. And children faced higher risks than adults. For different HMs, the carcinogenic risk order is Cr > Ni > As > Cd > Pb. Except for Cd, the carcinogenic risks via oral intake of Cr, Ni, As, and Pb are significantly higher than those via other exposure pathways.

## 4. Conclusions

The average concentrations of HMs in the surface sediments of river channels around the typical tailings area follow the order: Cu > Zn > Cr > Ni > Pb > As > Cd > Hg. Except for Cr, the average concentrations of the other HMs exceed the soil background values in Anhui Province. There are significant spatial differences in the distribution of HMs among different sampling points.

The pollution levels of Cd and Cu in the sediments are severe, with some sampling points reaching moderate-to-severe pollution levels. Both the single-factor pollution index and the Nemerow comprehensive pollution index indicate that heavy metal pollution in the upstream sediments of the Caishi River is relatively serious. The comprehensive ecological risk level of HMs at 85% of the sampling points reaches a high-risk level, with Cd, Cu, and Hg being the main ecological risk contributing factors. The human health risk assessment results show that both adults and children face carcinogenic risks from heavy metal exposure, with children having a higher carcinogenic risk than adults.

There is a significant positive correlation among different HMs in the sediments, suggesting a homogenous source of pollution. Principal component analysis indicates that the HMs in the river sediments of the study area are influenced by multiple sources, primarily agricultural fertilization and ore mining.

It is recommended to adopt measures of “external pollution interception + internal treatment” to manage heavy metal pollution in the river channels of the sampling area. Firstly, decommission the upstream tailings reservoir by removing tailings and re-greening the area with soil cover to reduce the sources of heavy metal pollution. Construct ecological ditches and artificial wetlands around the farmland near the river to reduce non-point source pollution. Secondly, implement dredging measures in high-risk areas of HMs in the river sediments and combine water ecological regulation technology to enhance the treatment effect.

## Figures and Tables

**Table 1:** Risk exposure parameters of heavy metal health risk assessment [25].

Parameters	Meaning	Unit	Reference Value of Adults	Reference Value of Children
Ring	Intake amount	mg/d	100	200
EF	Exposure frequency	d/a	345	345
ED	Exposure duration	a	24	6
CF	Conversion factor	Unitless	1.00 × 10^−6^	1.00 × 10^−6^
BW	Average body weight	kg	56.8	15.9
AT	Average carcinogenic exposure time	d	70 × 365	70 × 365
	Average non-carcinogenic exposure time	d	ED × 365	ED × 365
Rinh	Respiratory frequency	m^3^/d	14.5	7.5
PEF	Particle settling factor	m^3^/kg	1.36 × 10^9^	1.36 × 10^9^
SA	Skin exposure area	cm^2^	5700	2800
AF	Skin adhesion factor	mg/(cm^2^·d)	0.49	0.65
ABS	Skin absorption factor	Unitless	As: 0.03	
			Other heavy metal: 0.001	

**Table 2:** Reference dose (RFD) and slope factor (SF) for different exposure pathways of HMs [24].

Heavy Metal	RFD (mg/(kg·d))			SF		
	Oral	Dermal	Inhalation	Oral	Dermal	Inhalation
Cd	1.00 × 10^−3^	1.00 × 10^−5^	1.00 × 10^−5^			6.30
Cu	4.00 × 10^−2^	1.20 × 10^−2^	4.00 × 10^−2^			
Zn	3.00 × 10^−1^	6.00 × 10^−2^	3.00 × 10^−1^			
Cr	3.00 × 10^−3^	7.50 × 10^−5^	2.25 × 10^−5^	5.01	2.00	42.00
Pb	3.50 × 10^−3^	5.30 × 10^−4^	3.50 × 10^−3^	0.042		42.00
Ni	2.00 × 10^−2^	5.40 × 10^−3^	2.06 × 10^−2^	1.70	42.50	0.901
As	3.00 × 10^−4^	1.23 × 10^−4^	1.50 × 10^−5^	1.50	3.66	15.1
Hg	4.00 × 10^−4^	2.10 × 10^−5^	3.00 × 10^−4^			

**Table 3:** Summary results of HMs in sediments of the Caishi River.

Heavy Metals	Cd	Cu	Zn	Cr	Pb	Ni	As	Hg
Average (mg/kg)	1.07	348.24	180.67	47.19	28.85	42.03	11.80	0.10
Median (mg/kg)	0.94	254.26	166.47	50.55	29.87	39.91	10.36	0.10
Minimum (mg/kg)	0.07	36.70	67.53	21.56	15.26	19.91	3.16	0.02
Maximum (mg/kg)	2.35	965.34	321.09	61.16	52.10	67.09	29.65	0.18
Standard Deviation (mg/kg)	0.59	245.37	70.15	11.96	7.53	11.59	5.51	0.04
Coefficient of Variation	56%	70%	39%	25%	26%	28%	47%	44%
Soil Background Values in Anhui Province (mg/kg) [19]	0.10	24.90	53.20	69.40	25.90	25.00	9.40	0.04

**Table 4:** Mean values of carcinogenic and non-carcinogenic risk indices.

Risk Type	Heavy Metal	Adult				Child			
		Oral	Dermal	Inhalation	HI	Oral	Dermal	Inhalation	HI
Non-carcinogenic risk	Cd	1.78 × 10^−3^	4.97 × 10^−3^	1.90 × 10^−5^	6.77 × 10^−3^	1.27 × 10^−2^	1.16 × 10^−2^	3.51 × 10^−5^	2.43 × 10^−2^
	Cu	1.45 × 10^−2^	1.35 × 10^−3^	1.54 × 10^−6^	1.58 × 10^−2^	1.04 × 10^−1^	3.14 × 10^−3^	2.85 × 10^−6^	1.07 × 10^−1^
	Zn	1.00 × 10^−3^	1.40 × 10^−4^	1.07 × 10^−7^	1.14 × 10^−3^	7.16 × 10^−3^	3.26 × 10^−4^	1.97 × 10^−7^	7.49 × 10^−3^
	Cr	2.62 × 10^−2^	2.92 × 10^−2^	3.72 × 10^−4^	5.58 × 10^−2^	1.87 × 10^−1^	6.81 × 10^−2^	6.88 × 10^−4^	2.56 × 10^−1^
	Pb	1.37 × 10^−2^	2.53 × 10^−3^	1.46 × 10^−6^	1.62 × 10^−2^	9.80 × 10^−2^	5.89 × 10^−3^	2.70 × 10^−6^	1.04 × 10^−1^
	Ni	3.50 × 10^−3^	3.62 × 10^−4^	3.62 × 10^−7^	3.86 × 10^−3^	2.50 × 10^−2^	8.42 × 10^−4^	6.69 × 10^−7^	2.58 × 10^−2^
	As	6.55 × 10^−2^	1.34 × 10^−1^	1.40 × 10^−4^	1.99 × 10^−1^	4.68 × 10^−1^	1.04 × 10^−2^	2.58 × 10^−4^	4.78 × 10^−1^
	Hg	4.16 × 10^−4^	2.21 × 10^−4^	5.91 × 10^−8^	6.37 × 10^−4^	2.97 × 10^−3^	5.15 × 10^−4^	1.09 × 10^−7^	3.49 × 10^−3^
	THI	1.27 × 10^−1^	1.73 × 10^−1^	5.34 × 10^−4^	3.00 × 10^−1^	9.04 × 10^−1^	1.01 × 10^−1^	9.87 × 10^−4^	1.01
Carcinogenic risk	Cd			3.95 × 10^−6^				6.93 × 10^−6^	
	Cr	1.35 × 10^−4^	1.50 × 10^−6^	1.21 × 10^−7^	1.37 × 10^−4^	2.41 × 10^−4^	8.75 × 10^−7^	5.57 × 10^−8^	2.42 × 10^−4^
	Pb	6.91 × 10^−7^		7.37 × 10^−8^	7.65 × 10^−7^	1.23 × 10^−6^	0.00	3.40 × 10^−8^	1.27 × 10^−6^
	Ni	4.08 × 10^−5^	2.85 × 10^−5^	2.30 × 10^−9^	6.92 × 10^−5^	7.28 × 10^−5^	1.66 × 10^−5^	1.06 × 10^−9^	8.94 × 10^−5^
	As	1.01 × 10^−5^	6.88 × 10^−7^	1.08 × 10^−8^	1.08 × 10^−5^	1.80 × 10^−5^	4.01 × 10^−7^	5.01 × 10^−9^	1.84 × 10^−5^
	TCR	1.86 × 10^−4^	3.07 × 10^−5^	2.07 × 10^−7^	2.17 × 10^−4^	3.33 × 10^−4^	1.78 × 10^−5^	9.58 × 10^−8^	3.51 × 10^−4^

## Data Availability

The data presented in this study are available on request from the corresponding author due to (The data involved in this study are not publicly available due to the constraints of research project management regulations and the requirements for ongoing follow-up studies).

## Supplementary Materials

The following supporting information can be downloaded at: https://www.mdpi.com/article/10.3390/toxics14040284/s1, Table S1: The grades of geo-accumulation indexes; Table S2: Classes of RI and potential ecological risk factors; Table S3: Summary results of pH and in sediments of the Caishi River; Table S4: Pearson correlation analysis between pH, organic matter content, and heavy metal content [51].
