# Supplementary material for: Heavy Metals in Iron Tailing Around River Sediments of Xiangshan: Status, Risks, and Human Health Threats
Source: Toxics. 2026 Mar 27;14(4):284. doi: 10.3390/toxics14040284 (PMC13120278; doi:10.3390/toxics14040284)
Supplement: Supplementary file 1 [file toxics-14-00284-s001.zip › toxics-4209994-supplementary.pdf]

supporting materials for

Heavy Metals in Iron Tailing-Around Rivers Sediments of Xiangshan: Status, Risks and Human Health Threats

Chen Jun[1]，XIONG Guangcheng[2,3]，ZHANG Shutong[1]，Lv Xianghui[1], Tang Qiang[2,4], Zhou Qiuhong[5]

[1] China National Chemical Construction Investment Group Co.，Ltd，Beijing 102300；
[2] Changjiang Survey，Planning，Design and Research Co.，Ltd，Wuhan 430010;
[3] Hubei Provincial Engineering Research Center for Comprehensive Water Environment Treatment in the Yangtze River Basin, Wuhan 430010, China
[4] Key Laboratory of Yangtze River Management and Protection of Ministry of Water Resources，Wuhan 430010
[5] School of Resources and Civil Engineering, Gannan University of Science and Technology, Ganzhou 341000, Jiangxi, China

1. Single-Factor Pollution Index Method (SFPI)

The single-factor evaluation is based on the quality sub-index model, with the calculation formula as follows:

$$P_i = \frac{C_i}{S_i} \quad (1)$$

Where P_i represents the mass fraction of the pollutant factor; C_i is the measured *concentration of pollutant* factor *i*; *Si* is the reference value of pollutant factor *i*, using the background value of HMs in soil of Anhui Province in this study [19]. The P_i value is used to characterize the pollution degree of a single pollutant, defined as follows: $0.7<P_i<1$, safe; $0.7<P_i<1$, alert; $1\leq P_i<2$, slight pollution; $2\leq P_i<3$, moderate pollution; $P_i>3$, serious pollution.

2. Nemerow Pollution Index (NPI)

The NPI evaluates not only the impact of a single heavy metal on the environment but also the combined impact of multiple HMs. Thus, it is commonly used to assess the pollution status of HMs in soil and sediments. The calculation formula for the NPI is:

$$P_Z = \sqrt{\frac{(P_{imax})^2+(P_{iave})^2}{2}} \quad (2)$$

Where P_Z is the comprehensive pollution index of pollutants; P_{imax} is the maximum value of the single heavy metal pollution index; P_{iave} is the average value of the single factor index. When P_Z<0.7 is safe; 0.7<P_Z<1, alert; 1≤P_Z<2, slight pollution; 2≤P_Z<3, moderate pollution; P_Z>3, serious pollution [6].

3 Geo-accumulation Index Method (I_{geo})

The Geo-accumulation Index accounts for the background value of elements and is often used for the quantitative analysis of heavy metal pollution in sediments [20]. The calculation method for the Geo-accumulation Index is:

$$I_{geo}=\log_2(C_n/1.5B_n) \quad (3)$$

Where C_n is the concentration of heavy metal n in the sample; Bn is the geochemical background value of element n, which can be replaced by the background value of HMs in soil in the study area [19].

Table S1 The grades of geo-accumulation indexes

Geological Accumulation Index (Igeo)	Classification	Degree of Pollution
<0	0	Clean
0~1	1	Minor
1~2	2	Moderately slight
2~3	3	Moderate
3~4	4	Moderately severe
4~5	5	severe
5~10	6	Extreme severe

4. Potential Ecological Risk Index Method (PERI)

The PERI comprehensively considers the toxicity of HMs and the environmental response. It can reflect the impact of a single heavy metal element in sediments on the environment and the comprehensive effect of multiple HMs in a specific environment. The calculation formula is:

$$RI = \sum_{i=1}^{n} E_r^i = \sum_{i=1}^{n}(T_r^i \times C_f^i) \quad (4)$$

$$C_f^i = \frac{C_r^i}{C_0^i} \qquad (5)$$

Where *RI* represents the comprehensive potential ecological risk index of HMs in sediments, divided into 4 levels (Table 2); *n* is the number of heavy metal types; E_r^i is the potential ecological risk factor of single metal *r* (Table 2); C_f^i is the pollution coefficient; C_r^i and C_0^i are the content of heavy metal *r* in sediments and its reference value; T_r^i is the toxicity response factor of heavy metal element *r*, with toxicity response factors of Cd, Cu, Zn, Cr, Pb, Ni, As, and Hg being 30, 5, 1, 2, 5, 5, 10, and 40 respectively[1].

Table S2 Classes of RI and potential ecological risk factors

Single Heavy Metal		Multiple Heavy Metals	
E_r^i	Grading	RI	Grading
$E_r^i \le 40$	Low risk	RI≤150	Low risk
$40 < E_r^i \le 80$	Moderate risk	150<RI≤300	Moderate risk
$80 < E_r^i \le 160$	High risk	300<RI≤600	High risk
$160 < E_r^i \le 320$	Very high risk	600<RI	Very high risk
$320 < E_r^i$	Extremely high risk	/	/

5. Human Health Risk Assessment Model

Based on the migration and transformation characteristics of sediments in the surface environment, it is considered that sediment pollutants enter the human body mainly through the following three exposure pathways: direct ingestion via the hand-to-mouth route, dermal contact, and direct inhalation through the respiratory system. The calculation formulas are as follows:

Average daily intake dose (D_{ing}) via hand-to-mouth ingestion:

$$D_{ing} = C \times \frac{IngR \times EF \times ED}{BW \times AT} \times 10^{-6}$$

Daily average exposure via inhalation (Dinh)

$$D_{inh} = C \times \frac{InhR \times EF \times ED}{PEF \times BW \times AT}$$

Daily average exposure through skin contact (Ddermal)

$$D_{dermal} = C \times \frac{SL \times SA \times ABS \times EF \times ED}{BW \times AT} \times 10^{-6}$$

Lifetime average exposure to carcinogenic heavy metals in sediment (LADD):

$$LADD = \frac{C \times EF}{AT} \times \left(\frac{CR_{child} \times ED_{child}}{BW_{child}} + \frac{CR_{adult} \times ED_{adult}}{BW_{adult}} \right)$$

Where:

$IngR$ is the frequency of dust intake through the mouth; $InhR$ is the respiratory frequency; EF is the exposure frequency; ED is the duration of exposure; *SA* is the exposed skin surface area; *SL* is the skin adhesion; *PEF* is the particulate matter emission factor; *BW* is the average body weight; *AT* is the average exposure time; *ABS* is the skin absorption factor, dimensionless; *C* is the concentration of heavy metals at the exposure point ($mg \cdot kg^{-1}$); The *CR* values for different exposure routes are: *CR*=*IngR*, *CR*=*InhR*, *CR*=*SA*×*SL*×*ABS*.

Health risk characterization:

$$HQ = \frac{D}{RfD}$$

$$HI = \sum HQ_i$$

HQ is a non-carcinogenic risk quotient, which characterizes the non-carcinogenic risk of a single pollutant. When *HQ* is less than 1, the risk is considered to be low or negligible; when *HQ* is equal to 1, non-carcinogenic risk is considered to exist. *D* is the sum of D_{ing}, D_{inh}, and D_{dermal}; *RfD* is the reference dose, indicating the maximum amount of pollutant that can be ingested per unit of body weight per unit of time without causing adverse human reactions. *HI* represents the total non-carcinogenic risk from multiple exposure pathways[2-4].

$$Risk_i = LADD \times SF_i$$

$$Risk = \sum Risk_i$$

Risk refers to the carcinogenic risk, which is the probability of cancer occurrence; *SF* stands for the Slope Factor of Carcinogenesis, indicating the maximum probability of carcinogenic effects resulting from human exposure to a certain dose of a certain pollutant, expressed in mg/(kg·d).

6.

Table S3 Summary results of pH and in sediments of Caishi River

Heavy metals	pH	Organic matter(%)
Average	7.23	7.74
Median	7.3	7.72
Minimum	6.6	4.16
Maximum	7.7	14.5
Standard Deviation	0.35	2.58
Coefficient of Variation	4.82%	33.3

7. Correlation analysis between sediment pH, organic matter content, and heavy metal content in sediment

Table S4 Pearson correlation analysis between pH, organic matter content, and heavy metal content

HMs	OM	pH
Cd	-0.01	0.53
Cu	0.19	-0.28
Zn	0.01	0.57
Cr	0.20	-0.24
Pb	0.07	0.06
Ni	0.27	0.05
As	0.40	-0.41
Hg	0.20	-0.09
pH	-0.17	

1. Hakanson, L., An ecological risk index for aquatic pollution control. A sedimentological approach. *Water research* **1980,** 14, (8), 975-1001.
2. Agency, U. S. E. P., Integrated Risk Information System (IRIS). In U.S. Environmental Protection Agency: Washington, D.C., 2011.
3. Agency, U. S. E. P., Regional Screening Levels (RSLs) for Chemical Contaminants; U.S. EPA. In U.S. Environmental Protection Agency: Washington, D.C., 2014.
4. Council, N. R.; Studies, B. o. E.; Process, C. t. R. t. I., Review of EPA's integrated risk information system (IRIS) process. **2014**.
